# Chemical Investigation of Household Solid Fuel Use and Outdoor Air Pollution Contributions to Personal PM_2.5_ Exposures

**DOI:** 10.1021/acs.est.1c01368

**Published:** 2021-11-24

**Authors:** Alexandra Lai, Martha Lee, Ellison Carter, Queenie Chan, Paul Elliott, Majid Ezzati, Frank Kelly, Li Yan, Yangfeng Wu, Xudong Yang, Liancheng Zhao, Jill Baumgartner, James J. Schauer

**Affiliations:** † Environmental Chemistry and Technology Program, University of Wisconsin-Madison, Madison, Wisconsin 53706, United States; ‡ Department of Epidemiology, Biostatistics, and Occupational Health, McGill University, Montreal, Quebec H3A 1A3, Canada; § Department of Civil and Environmental Engineering, Colorado State University, Fort Collins, Colorado 80523, United States; ∥ MRC Centre for Environment and Health, Department of Epidemiology, Biostatics, and Occupational Health, School of Public Health, Imperial College London, London W2 1PG, U.K.; ⊥ Department of Analytical, Environmental, and Forensic Sciences, Kings College London, London SE1 9NH, U.K.; # Clinical Research Institute, Peking University, Beijing 100191, China; ¶ Department of Building Science, Tsinghua University, Beijing 100084, China; ∇ Fuwai Hospital, Chinese Academy of Medical Sciences, and Peking Union Medical College, Beijing 100037, China; ○ Institute for Health and Social Policy, McGill University, Montreal, Quebec H3A 1A3, Canada; ⧫ Wisconsin State Laboratory of Hygiene, University of Wisconsin-Madison, Madison, Wisconsin 53718, United States

**Keywords:** personal exposure, household air pollution, solid fuels, molecular tracers, personal/outdoor ratio, PM_2.5_, source apportionment

## Abstract

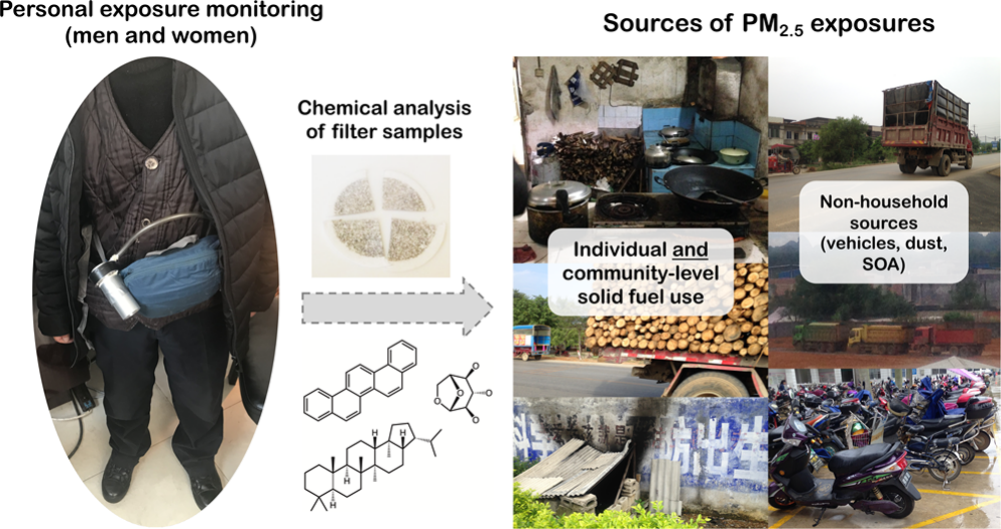

In communities with household solid fuel use, transitioning to clean stoves/fuels often results in only moderate reductions in fine particulate matter (PM_2.5_) exposures; the chemical composition of those exposures may help explain why. We collected personal exposure (men and women) and outdoor PM_2.5_ samples in villages in three Chinese provinces (Shanxi, Beijing, and Guangxi) and measured chemical components, including water-soluble organic carbon (WSOC), ions, elements, and organic tracers. Source contributions from chemical mass balance modeling (biomass burning, coal combustion, vehicles, dust, and secondary inorganic aerosol) were similar between outdoor and personal PM_2.5_ samples. Principal component analysis of organic and inorganic components identified analogous sources, including a regional ambient source. Chemical components of PM_2.5_ exposures did not differ significantly by gender. Participants using coal had higher personal/outdoor (P/O) ratios of coal combustion tracers (picene, sulfate, As, and Pb) than those not using coal, but no such trend was observed for biomass burning tracers (levoglucosan, K^+^, WSOC). Picene and most levoglucosan P/O ratios exceeded 1 even among participants not using coal and biomass, respectively, indicating substantial indirect exposure to solid fuel emissions from other homes. Contributions of community-level emissions to exposures suggest that meaningful exposure reductions will likely require extensive fuel use changes within communities.

## Abstract 2

Exposure to household air pollution depends not only on an individual’s own fuel use but also on outdoor air pollution and their neighbors’ fuel use.

## Introduction

Approximately 3.6 billion people worldwide and 500 million people in China primarily cook with stoves that burn solid fuels such as coal and biomass.^1^ Household solid fuel burning emits high levels of pollutants including fine particulate matter (PM_2.5_) into homes and communities, contributing to high levels of exposure to household air pollution (HAP). Cooking area concentrations of PM_2.5_ can exceed the World Health Organization’s 24 h guideline level of 25 μg/m^3^ by an order of magnitude or more, contributing to personal PM_2.5_ exposures both directly within the household and indirectly via contributions to ambient PM_2.5_.^2^ Exposure to PM_2.5_ is associated with an increased risk of adverse health outcomes across the life course, including cardio-respiratory diseases,^3,4^ low birth weight,^5^ and diabetes,^6^ thereby motivating the development of diverse strategies to reduce PM_2.5_ and improve public health.^1^

Identifying the sources contributing to PM_2.5_ exposures is essential for developing more effective and appropriately targeted mitigation strategies. Transitioning from solid fuels to less-polluting household energy systems can significantly lower HAP;^7^ however, field studies show that even with use of cleaner fuels, PM_2.5_ exposures often remain above the WHO guideline.^2,7−9^ Achieving and sustaining low exposures to PM_2.5_ among populations using solid fuels requires both household-level changes in energy practices and community- and regional-level mitigation of external PM sources, including industry, traffic, agricultural burning, and HAP from neighboring households.^9^

Personal monitoring of airborne PM is intended to account for variability in PM exposures that are attributable to variability in individual behaviors and living conditions.^7^ Technological developments in the size, weight, noise, and cost of personal air samplers contributed to an increase in direct sampling of personal PM_2.5_ exposures in settings with HAP over the last decade.^10−29^ These studies showed high within-subject variability in exposures and relatively small differences between clean fuel and solid fuel users, which could be driven by diverse factors including outdoor air pollution, unidentified sources, variability in emissions based on fuel characteristics or stove performance, and solid fuel use in nearby homes.^2,9^ Chemical analysis of PM_2.5_ samples could provide information about the relative impacts of these factors on exposure reductions or lack thereof; however, only a small handful of these studies included chemical analysis^10−12,21−24,30^ and even fewer measured source-specific tracers (e.g., levoglucosan).^10,23,24,30^

Using data from the INTERMAP China Prospective (ICP) study, a geographically diverse cohort of men and women with different fuel use patterns and outdoor source contributors, we investigated outdoor and personal exposures to PM_2.5_, its chemical components, and source contributions. Our specific objectives were to (1) measure and compare the chemical composition and sources of PM_2.5_ exposures by region, gender, and predominant fuel use patterns and (2) differentiate between direct and indirect (not from that individual’s home) exposures to PM_2.5_ emitted by solid fuel combustion. In this study, we employed chemical analysis and source apportionment techniques paralleling our previous analyses of PM_2.5_ exposures in rural China^24^ but with additional dimensions: diversity of fuel types (i.e., coal, biomass, and clean fuels such as gas and electricity) and use, inclusion of both men and women, and multiple study sites. Understanding the composition and sources of PM_2.5_ exposures is important for refining exposure assessments, which in turn are essential for designing effective interventions and mitigation strategies.^7,9,31^

## Methods

### Study Design

The PM_2.5_ samples analyzed in this study were collected for the ICP study, a longitudinal study in three provinces in northern (Beijing and Shanxi) and southern China (Guangxi) established to identify environmental and dietary risk factors for cardiovascular disease. The ICP study design, participants, and data collection have been described elsewhere.^32−34^ Briefly, 787 individuals aged 40–79 were enrolled in the ICP study (258 in Beijing, 290 in Shanxi, and 239 in Guangxi) in 2015 and 2016. All three study sites were rural but becoming more peri-urban, and households used biomass and/or liquefied petroleum gas (LPG) for cooking. At the Shanxi and Beijing sites, coal was also used for cooking and/or indoor space heating in winter. Through a visual questionnaire, participants recorded their use of different stove and fuel types for cooking and, where applicable, heating. Study sites are described in more detail in Section S1 and fuel use data collection and trends in Section S2 and Figure S2. Participants provided written informed consent. Study protocols were approved by ethical review boards at all investigator institutions.

### Air Pollution Sampling

Personal exposure PM_2.5_ samples were collected for 24-h sampling periods on 37 mm polytetrafluoroethylene (PTFE) membrane filters (Zefluor, Pall Corporation, USA), using Harvard personal exposure monitors (PEMs) (Mesa Laboratories, USA).^35^ PEMs, which include impactors for PM_2.5_ size selection, were attached to personal sampling pumps (Apex Pro and TUFF, Casella Inc.; USA) operating at 1.8 L/min. Participants carried PEMs with them in a waistpack except while bathing or sleeping, when they kept the PEM nearby and off the ground. Pedometers were also included in waistpacks to monitor compliance, where samples were flagged as potentially noncompliant and excluded from these analyses if they had step counts of <500 steps.^32^

Outdoor PM_2.5_ samples were also collected for 24-h intervals on 37 mm Zefluor PTFE filters, placed inside either PEMs or cassettes paired with cyclones (Mesa Laboratories, USA) attached downstream from sampling pumps operating at 1.8 or 3.5 L/min, respectively. Outdoor monitors were positioned at least 4 m from the ground in a location that was central to each study village (Shanxi; *n* = 6 villages) or the group of study villages (Beijing and Guangxi), at least 30 m from a household chimney, and at least 100 m from other known PM_2.5_ sources including local industry and major roadways. We collected 10% field blanks. Pump flow rates were checked at the start and end of each sampling period against a rotameter, which was calibrated at the start and end of each field campaign using a primary gas flow meter as described in Lee et al. (2021).^32^

Filters were weighed before and after sampling using an automatic weighing system (Mettler-Toledo, USA) in a temperature- and humidity-controlled environment.^24,36^ The weighing room maintained a 24-h average temperature of 20–23 °C (standard deviation ≤ 2 °C) and a 24-h average relative humidity of 30–40% (standard deviation ≤ 5%). Filters were equilibrated for 24 hours before being weighed, and the reported weights were the average of duplicate measurements with differences <5 μg. Detailed information on air pollution measurement and related quality control procedures are published elsewhere.^32^

### Sample Compositing for Chemical Analysis

For chemical analyses, we selected a subset of 221 PM_2.5_ exposure samples and 26 outdoor PM_2.5_ samples (out of 2073 personal and 48 outdoor PM_2.5_ samples). Within this subset, each exposure sample had a corresponding outdoor sample collected on the same day at the same study site (Figure S1). Fewer outdoor samples were required because personal sampling was conducted with multiple participants in parallel: for each outdoor sample, several personal exposure samples were collected concurrently at that site. All selected samples were collected in winter (November/December 2015 and 2016), and personal exposure samples were from current nonsmokers. Each filter was divided into sections for different chemical analyses. To obtain sufficient mass, filter sections were combined into 43 personal exposure and 5 outdoor composites (Figure S1, Table 1). Correspondence between outdoor and personal was preserved in the compositing scheme. For example, the Guangxi outdoor composite had samples collected on seven sampling dates, and each of the Guangxi personal exposure composites had samples from those same seven dates. Composite fuel use was defined based on whether each fuel was used in any of the individual samples; samples with similar fuel use were grouped together in composites when possible. Sample selection, compositing, and fuel use are described in further detail in Section S2 and Figure S2.

**Table 1:** Summary of Samples Selected for Chemical Analysis Composites [Composites (Individual Samples)]^a^

	sample type		
study site	personal exposure, women	personal exposure, men	outdoor
Guangxi	5 (35)	2 (14)	1 (7)
Beijing	11 (55)	9 (45)	2 (10)
Shanxi	11 (49)	5 (23)	2 (9)

a Data are presented as *N* (*M*) where *M* is the number of individual samples that were combined to form *N* composites for chemical analysis.

Eleven field blank composites (of 58 individual field blank filters) were also analyzed, corresponding to sample sites and the number of individual filters per composite. The majority of the field blanks were also from winter sampling seasons, with a small number of summer field blanks included when winter field blanks were not available. Seasonal differences in field blank filter masses were not statistically significant and were comparable to between-site differences, which were accounted for in the blank compositing scheme (Figure S3).

### Chemical Analyses

The following chemical species were measured: water-soluble organic carbon (WSOC), water-soluble ions, trace and major elements, and organic molecular markers. Samples were divided into one half and two quarter sections before compositing for separate chemical analyses (Figure S1). Chemical analyses were conducted according to previously published methods^24,37−39^ and summarized below.

Composites of quarter filter sections for WSOC/ion analysis were extracted in 15 mL of ultrapure water on a shaker table for 6 hours and then filtered through a polypropylene syringe filter (0.45 μm pore; Whatman). A total organic carbon analyzer (M9 TOC Analyzer, Sievers/GE) was used to measure WSOC, and seven water-soluble ions (SO_4_^2–^, NO_3_^–^, Cl^–^, Na^+^, NH_4_^+^, K^+^, and Ca^2+^) were measured using ion chromatography (IC) (Dionex ICS 1100/2100, Thermo Fisher Scientific).

After microwave digestion of elemental analysis composites (quarter sections) in ultrapure nitric acid, total concentrations of 51 elements (Table S1) were quantified using magnetic sector ICPMS (Thermo-Finnigan Element 2, Thermo Fisher Scientific).

Organic molecular marker composites (half sections) were extracted by Soxhlet in 50:50 dichloromethane/acetone and then concentrated with a rotary evaporator and ultrapure nitrogen gas. Organic compound standards were added to each sample,^38^ and each batch of ∼10–12 samples included a laboratory blank and a blank spiked with either standards or 10 mg of standard reference material (NIST SRM 1649, Urban Dust), extracted using the same procedure as the samples. Compound classes analyzed included polycyclic aromatic hydrocarbons (PAHs), hopanes and steranes (markers of fossil fuel combustion), *n*-alkanes, *n*-alkanoic acids, aliphatic and aromatic acids, and levoglucosan.^24^

### Data Analysis

Source contributions to PM_2.5_ were estimated using chemical mass balance (CMB) to apportion primary source contributions and mass reconstruction for secondary inorganic aerosol (SIA) and dust. The CMB model calculates source contributions to measured concentrations of chemical species in PM_2.5_ by solving a system of linear equations using an effective variance-weighted least-squares method.^40^ We input the following source tracers: levoglucosan, picene, 17α(H)-21β(H)-30-norhopane, 17α(H)-21β(H)-hopane, sulfate, nitrate, and ammonium. The sources considered were residential wood burning and bituminous coal combustion, sampled in typical Chinese cooking and heating stoves,^41,42^ and mixed diesel/gasoline vehicle emissions sampled from Zhujiang Tunnel in Guangzhou, China.^43^ Chemical profiles of these sources have been published previously.^24,41^*R*^2^ values were above 0.98 and χ^2^ (chi-square) values were below 1.9, in agreement with recommended parameters for the model (*R*^2^ > 0.8 and χ^2^ < 4).^44^ SIA was defined as the sum of sulfate, nitrate, and ammonium after correcting for primary source contributions to each.^24^ Dust mass was calculated as the sum of oxides of major crustal elements: 2.14*[Si] + 1.89*[Al] + 1.43*[Fe] + 1.40*[Ca] + 1.66*[Mg] + 1.67*[Ti] + 1.58*[Mn].^45,46^ Si concentrations were estimated using Al concentrations and typical crustal ratios.^47^

Principal component analysis (PCA) was conducted using the “psych” package (version 2.0.7) in the statistical software R (version 4.0.2) (https://www.r-project.org). Varimax rotation was applied to maximize factor loadings, and five principal components were selected on the basis of eigenvalues (all eigenvalues >1), variance explained (each >10% of total variance), and interpretability.

## Results and Discussion

### PM_2.5_ Concentrations and Chemical Components

**Figure 1:**
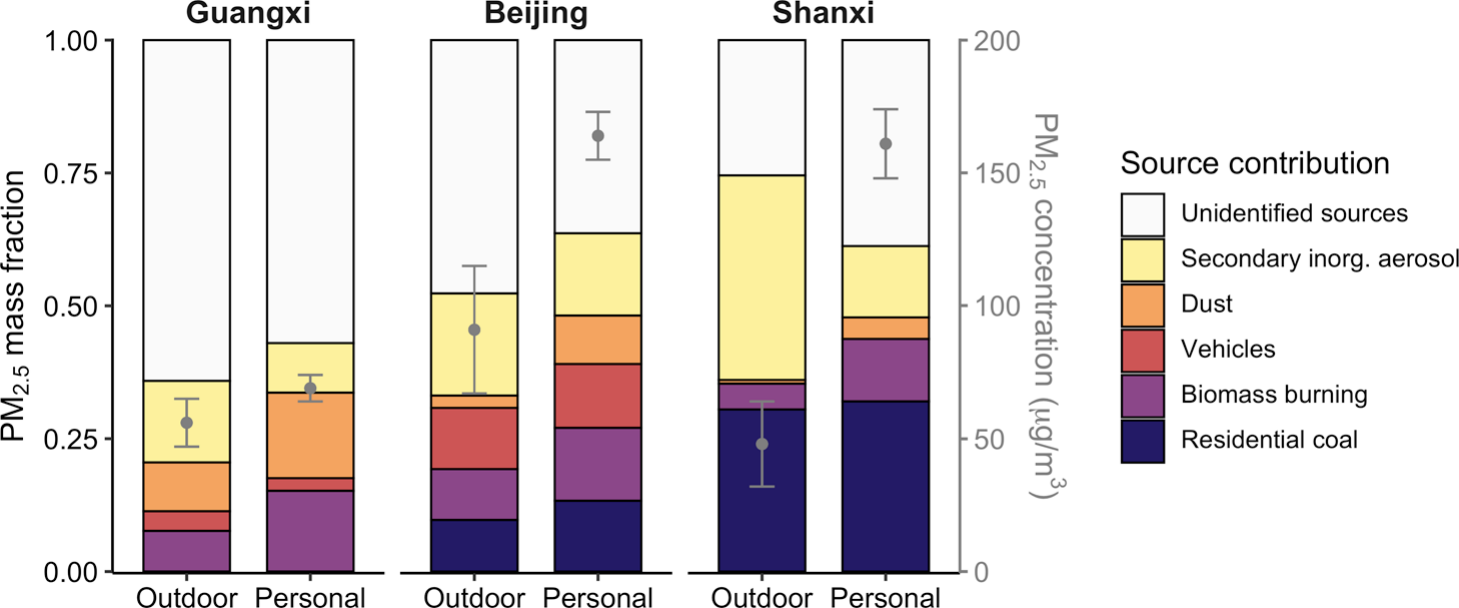
Average source contributions to ambient PM_2.5_ and personal PM_2.5_ exposures at each study site, normalized to PM_2.5_ mass. “Unidentified sources” refer to the difference between gravimetric PM_2.5_ and the sum of identified source contributions from the CMB model. Gray points and error bars represent the average and standard error, respectively, of PM_2.5_ concentrations for each site/sample combination and are plotted according to the gray secondary *y*-axis on the right.

Results for all personal PM_2.5_ exposure and outdoor PM_2.5_ measurements are presented and discussed in Lee et al. (2021).^32^ Here, for context and comparison with the overall data set, we briefly summarize the PM_2.5_ mass data for the samples selected for chemical analyses. Outdoor PM_2.5_ levels were the highest for the Beijing samples (arithmetic mean ± standard error: 91 ± 24 μg/m^3^), while Shanxi and Guangxi had similar outdoor PM_2.5_ (48 ± 16 and 56 ± 9 μg/m^3^, respectively) (Figure 1). Personal PM_2.5_ exposures were similar between men and women at each site and were higher for Shanxi (men: 168 ± 19 and women: 158 ± 17 μg/m^3^) and Beijing samples (men: 157 ± 13 and women: 171 ± 13 μg/m^3^) than for Guangxi samples (men: 71 ± 7 and women: 68 ± 6 μg/m^3^) (Figure S4). There are two likely reasons why exposures were lower among the Guangxi samples: the selected samples were collected in winter, when indoor space heating was necessary at the Shanxi and Beijing study sites but not Guangxi, and there was no coal use in the Guangxi households (Figure S2). Mass concentrations are representative of the samples overall: within each study site and sample type (outdoor and personal), median PM_2.5_ in the chemical analysis subset was within 25% of the median of all samples (Figure S5).

Among the samples selected for chemical analysis, average outdoor and personal PM_2.5_ exposures in Beijing and personal exposures in Shanxi exceeded the China National PM_2.5_ standard (75 μg/m^3^).^48^ All outdoor and personal exposure composites had average PM_2.5_ concentrations exceeding the World Health Organization’s 24 h PM_2.5_ guideline (Figure S4).^49^ Although indoor solid fuel use is highlighted as a major contributor to PM_2.5_ exposures, ambient air pollution in rural areas is not negligible.^50,51^ Throughout China between 2001 and 2015, average PM_2.5_ levels in rural areas were only about 5–20 μg/m^3^ lower (up to ∼30%) than in the corresponding urban areas of each province.^52^

Concentrations of PAHs, elements, ions, and WSOC are summarized (mean ± SD) in Table S1. Concentrations of K, emitted from biomass burning as K^+^,^53^ in our study sites (mean: 857, 1267, and 677 ng/m^3^ in Guangxi, Beijing, and Shanxi, respectively) were similar to those measured in a recent study of chemically speciated PM_2.5_ exposures among rural Chinese adults using both biomass and clean fuel stoves (mean: 691 and 1678 ng/m^3^ in agricultural and nomadic villages, respectively).^12^ By comparison, exposures to elements associated with coal combustion^54^ in our northern study sites (Beijing: 7.9 ng/m^3^ As and 96 ng/m^3^ Pb; Shanxi: 10.3 ng/m^3^ As and 113 ng/m^3^ Pb) were much higher than those reported by Ye et al. (As was not detected in most exposures, <5 ng/m^3^ Pb at both agricultural and nomadic villages), consistent with no documented coal use at their study site.^12^

Average concentrations of benzo[*a*]pyrene, a carcinogenic and commonly measured PAH, were 1.2, 20.1, and 12.8 ng/m^3^ in Guangxi, Beijing, and Shanxi, respectively. These levels were on the lower end of reported mean benzo[*a*]pyrene exposures (in ng/m^3^) among biomass (∼18–96^12,22,23,30^), coal (15–83^21−23^), and mixed (12–20^21,23^) biomass/coal users in rural China but exceeded urban benzo[*a*]pyrene exposures measured in Hong Kong, where study participants cooked only with gas or electricity (mean: 0.02 ng/m^3^).^55^

### Sources of Outdoor and Personal PM_2.5_

Coal combustion was the dominant primary source in Shanxi outdoor samples, contributing 16–41% of outdoor PM_2.5_, whereas the biomass burning contribution was smaller (5%) (Figure 1, Table S2). Coal and biomass contributions were similar in Beijing (8–11 and 6–13%, respectively). In Guangxi, biomass contributed approximately 8% of outdoor PM_2.5_, and no coal signature was detected. Motor vehicles were among the largest contributors to outdoor PM_2.5_ in Beijing (12%), which was similar to the solid fuel contribution, whereas vehicles comprised very little outdoor PM_2.5_ in Guangxi (2%) and were not identified as a source in Shanxi. Outdoor dust levels were the highest in Guangxi, in terms of both absolute (5.2 μg/m^3^) and mass-normalized concentrations (9%), and negligible in Beijing and Shanxi. SIA was the largest component of outdoor PM_2.5_ at all sites. Unidentified sources (mass not apportioned by the CMB model) include secondary organic aerosol (SOA) formation,^56,57^ which is supported by the results of the PCA discussed below. On a mass basis, outdoor PM_2.5_ sampled in this study was highly correlated with measurements from nearby government air-monitoring stations, indicating that these samples are representative of regional PM_2.5_.^32^

On a regional scale, source apportionment studies in China report that household solid fuel combustion is a major source of ambient PM_2.5_.^58,59^ Biomass and coal combustion have also been identified as major sources of volatile organic compounds (i.e., potential SOA precursors) in ambient PM,^60−65^ including rural or background sites in China.^60,63−65^ Solid fuel combustion contributed 8–46% of outdoor PM_2.5_ in this study in terms of primary emissions and thus likely also contributed to SOA.

Sources and components of personal exposure PM_2.5_ resembled outdoor PM_2.5_ in terms of mass fractions, suggesting substantial influence, and were generally higher than outdoor in terms of absolute concentrations, with the exception of SIA (Figure 1). Biomass burning and dust were the two major personal exposure sources in Guangxi, contributing 8–22 and 7–29% of PM_2.5_ exposures, respectively. Although picene was not detected in any of the Guangxi samples and thus coal contributions were considered to be zero, sulfate was the most abundant secondary ion in Guangxi personal exposure PM_2.5_ (6–9% was sulfate, compared with 3% or less nitrate or ammonium), indicating possible long-range transport of coal emissions including secondary sulfate precursors.^66,67^ Biomass, coal, and vehicle emissions contributed approximately equally to exposures in Beijing, with median contributions of 10–12% of PM_2.5_, ranging from 6 to 39% from biomass and 7–27% from coal combustion and vehicles. Dust contributed 5–17% of Beijing personal exposures, and SIA concentrations were highly variable, with median contributions of 1–6% but maxima up to ∼30%. In Shanxi, coal was the dominant source of exposures to PM_2.5_, contributing on average 32% (median: 29%), but as high as 86%, of PM_2.5_ mass. Biomass burning accounted for up to 25% of Shanxi PM_2.5_ exposures, and dust and secondary ions generally contributed less than ∼10%. In addition to SOA, unapportioned personal exposure PM_2.5_ mass also includes contributions from sources related to personal activity such as household dust.^24^

As another approach to identify major sources, we conducted a PCA using both organic and inorganic chemical components (Table 2). The CMB and mass reconstruction approach described above (Figure 1) is useful for quantifying primary source contributions, particularly in small data sets, but is limited because it requires input of source profiles and does not quantify secondary source contributions.^40^ PCA is an exploratory data analysis technique that does not require detailed knowledge of contributing factors/sources and can be applied to smaller data sets than multivariate source apportionment techniques such as positive matrix factorization (PMF) and thus is applicable in this context to supplement CMB and provide more information about PM_2.5_ sources.

**Table 2:** PCA Results

	principal component name	variance explained (%)	determining species (>0.8, 0.6–0.8, 0.4–0.6)^a^
PC1	coal combustion	22	**As**, Pb, *SO*_*4*_^*2–*^, *picene*, *BbF*, BeP, Zn
PC2	regional aerosol	19	**IcdP**, NO3–, *NH*_*4*_^+^, *hopane*, *ΣBTCAs*, norhopane, BbF
PC3	roadway emissions	17	**Fe**, Mn, Ba, Zn, norhopane, hopane
PC4	soil dust	11	**Ti**, **ΣREEs**, Ca, Ba
PC5	biomass burning	10	**WSOC**, wsK^b^, levoglucosan

a Species for each principal component are listed in order of loading from highest to lowest; those with loadings >0.8 in bold, between 0.6 and 0.8 in italics, and between 0.4 and 0.6 in regular font. BbF: benzo[*b*]fluoranthene; BeP: benzo[*e*]pyrene; IcdP: indeno[1,2,3-*cd*]pyrene; ΣBTCAs: sum of benzenetricarboxylic acids (1,2,3-, 1,2,4-, and 1,3,5-); norhopane: 17α(H)-21β(H)-30-norhopane; hopane: 17α(H)-21β(H)-hopane; and ΣREEs = sum of rare-earth elements (La, Ce, Pr, Nd, Sm, Eu, Dy, Ho, Yb, and Lu).

b Water-soluble K (wsK) was calculated by subtracting K^+^ (measured by IC) from total K (measured by ICPMS).

The sources identified using PCA were consistent with those used in CMB and mass reconstruction, with some differences that help to explain the “unidentified sources”. The component explaining the most variance, PC1, likely represents coal combustion: As and Pb, metals found in emissions from coal combustion as well as coal fly ash,^68^ had the highest loadings. Other determining species in PC1 are also known tracers of coal combustion: sulfate,^66,67^ zinc,^68,69^ and particularly picene, a PAH specific to coal combustion emissions.^41,54^

Nitrate, ammonium, hopanes, benzenetricarboxylic acids, and PAHs were the determining species for PC2, suggesting a mixed regional aerosol source (including secondary aerosols). Ammonium nitrate is a major component of SIA, and the presence of hopanes and PAHs suggests precursor sources of vehicles/coal and other combustion, respectively.^70^ Benzenetricarboxylic acids are thought to be derived from aromatic compounds such as PAHs and have been correlated with SOA in ambient aerosols.^71,72^ In source apportionment studies utilizing both CMB and a multivariate method such as PMF, PMF secondary aerosol source contributions have corresponded well with mass not apportioned by CMB,^56,57^ supporting our hypothesis that “unidentified sources” include secondary aerosol.

The elements with higher loadings in PC3—Fe, Mn, Ba, and Zn—have as common sources brake wear, tire wear, and/or road dust.^73−75^ Along with the presence of hopanes, this indicates that PC3 likely represents a roadway source comprising both tailpipe and non-tailpipe emissions.^75^ We included vehicle tailpipe emissions in the CMB model, but non-tailpipe emissions are derived from multiple sources (e.g., brake wear and tire wear) with variable inorganic and organic content,^75,76^ making them ill-suited to include in the CMB model. Predominance of the crustal elements Ti, Ca, and summed rare-earth elements in PC4 suggests that PC4 represents resuspended soil dust.^73^ PC5 is dominated by levoglucosan, water-soluble K, and WSOC, all of which are tracers of biomass burning emissions.^77^

### Personal/Outdoor Ratios of Chemical Components

We calculated personal-to-outdoor concentration ratios, using each personal exposure composite’s corresponding outdoor composite from the same sampling dates and site, in order to investigate the sources of personal exposures in relation to outdoor air pollution.

**Figure 2:**
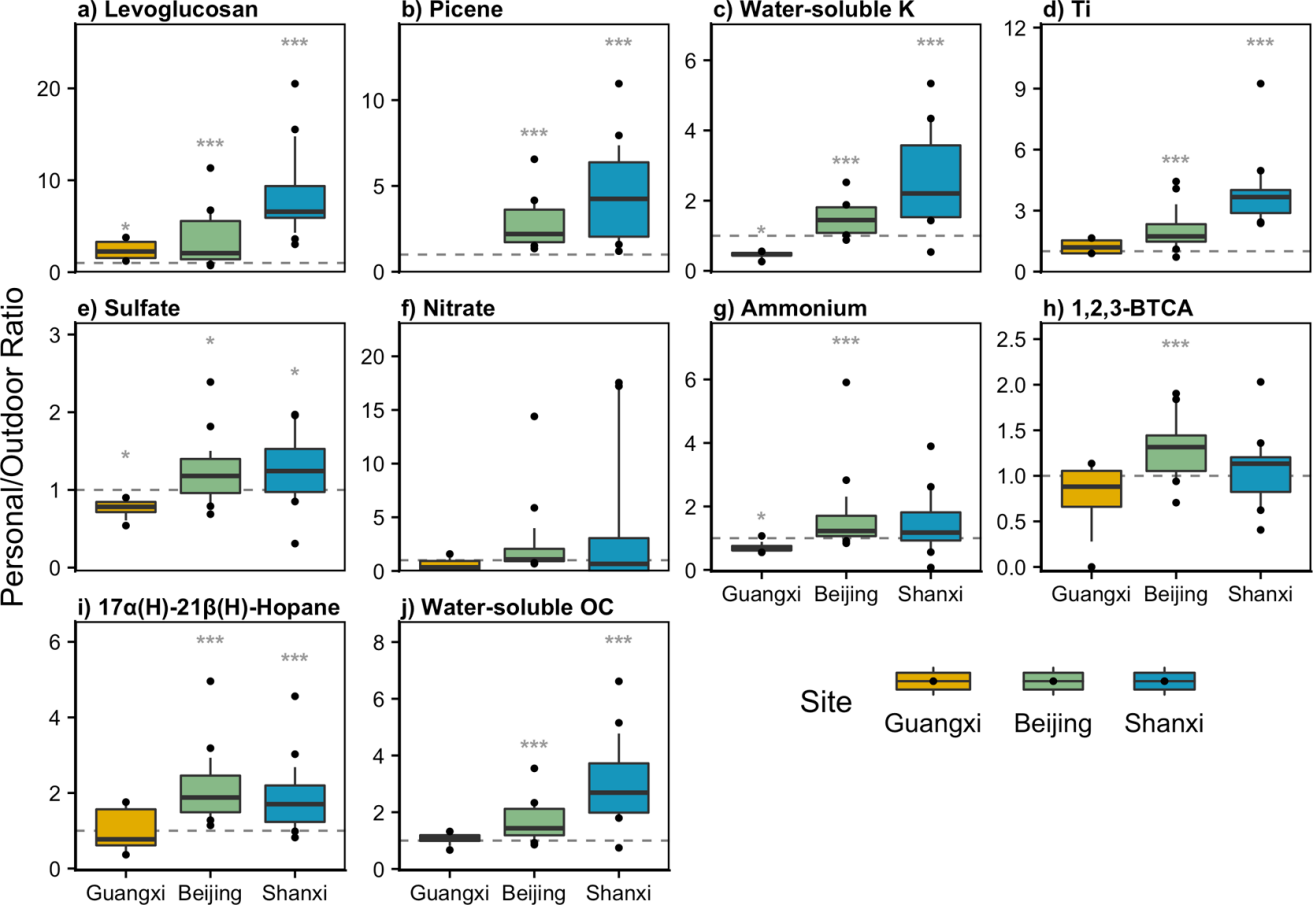
Personal/outdoor ratios of selected chemical components of PM_2.5_. The dashed line is plotted at *y* = 1 for reference. 1,2,3-BTCA = 1,2,3-benzenetricarboxylic acid. Box midlines indicate median ratios, the box represents the interquartile range (IQR; top = 75th percentile and bottom = 25th percentile), and whiskers extending above and below the box mark the 90th and 10th percentiles, respectively. Individual points represent values that were above the 90th percentile or below the 10th percentile. No data are plotted for picene in Guangxi because it was not detected in outdoor or personal exposure PM_2.5_ samples there. Gray asterisks denote statistical significance (one-sample Wilcoxon test; *** represents *p* < 0.001, ** represents *p* < 0.01, and * represents *p* < 0.05).

Primary tracers of solid fuel combustion (levoglucosan, picene, and water-soluble K)^53,54^ and dust (Ti)^73^ were enriched in the majority of personal PM_2.5_ exposure samples (Figure 2a–d). Levoglucosan was up to 20 times higher in personal exposures than in outdoor PM, with median P/O ratios of 2.3, 2.1, and 6.6 in Guangxi, Beijing, and Shanxi, respectively. Outdoor levoglucosan concentrations were the lowest in Shanxi (Table S1), driving the higher P/O ratios observed. For comparison, P/O ratios were much lower (0.5–1.5) among individuals without reported household use of solid fuels in Guangzhou.^78^ Picene, a marker of coal combustion, was not detected in personal or outdoor samples in Guangxi and was elevated in all Beijing and Shanxi personal exposures. Fuel use data indicate that the Guangxi personal exposure composites did not include any participants using coal, while all composites in Shanxi and the majority in Beijing did include coal use (Figure S2), which corresponds well to the observed picene trend. A nationwide household energy survey reported that, at the province level in rural communities, Guangxi had lower coal use (2.8%) than Beijing (5.8%) or Shanxi (58.6%).^79^ Water-soluble K was most enriched in personal exposures in Shanxi, slightly enriched in Beijing exposures, and lower in personal PM than in outdoor PM in Guangxi. This trend is consistent with major outdoor contributions to exposure: biomass was used in the majority of Guangxi composites but only half of the Shanxi composites, and relative biomass contributions to outdoor PM were higher in Guangxi than in Shanxi (Figure 1).

Titanium, a crustal element representative of total dust, had the lowest P/O ratios in Guangxi (1.2), slightly elevated P/O ratios in Beijing (1.7), and the highest in Shanxi (3.7). This may be driven by outdoor PM: outdoor dust concentrations were higher in Guangxi than at the other two sites, and average personal exposure dust concentrations were similar across sites (7–15%). Dust exposures exceeding the corresponding indoor or outdoor dust concentrations have previously been observed in rural settings,^11,24^ and dust is a plausible component of the “personal cloud” effect, where individuals’ behavior acts as an additional source of exposure.^80,81^

The P/O ratios of species associated with secondary aerosol were below or approximately one, indicating that these tracers are generally not present above outdoor levels in personal PM_2.5_ exposures. Median P/O ratios of SO_4_^2–^, NO_3_^–^, and NH_4_^+^, which comprise SIA, were 0.4–0.8 in Guangxi, 1.1–1.2 in Beijing, and 0.7–1.2 in Shanxi (Figure 2e–g). As noted earlier in discussion of the PCA, benzenetricarboxylic and other aromatic acids are candidate tracers for anthropogenic SOA. A representative aromatic acid, 1,2,3-benzenetricarboxylic acid, was not enriched in personal exposures: as with the secondary ions, median P/O ratios were 0.9–1.3 at all three sites (Figure 2h). In contrast to solid fuel combustion, secondary aerosol exposures are only due to outdoor air pollution, and P/O ratios close to or less than 1 for secondary aerosol tracers are consistent with that.

Trends in the 17α(H)-21β(H)-hopane and WSOC (Figure 2i,j) were more complex. Hopane concentrations were somewhat elevated in Beijing and Shanxi personal exposures but not in Guangxi (median P/O: 1.9, 1.7, and 0.8, respectively). In an urban context, hopanes are predominantly tracers of vehicle combustion, but with prevalent household coal combustion in the Beijing and Shanxi study areas, coal combustion is also a likely source.^41^ Hopane/picene ratios and hopane concentrations in general were higher in Beijing than in Shanxi (Figure S6). Given the specificity of picene as a tracer of coal combustion,^54^ hopane levels are likely more influenced by coal in Shanxi than in Beijing. WSOC was the most elevated in Shanxi (P/O = 2.7), slightly enriched in Beijing exposures (P/O = 1.4), and similar to outdoor in Guangxi personal exposures (P/O = 1.1). Biomass burning and SOA are typically considered the main sources of atmospheric WSOC. However, levoglucosan emission rates and OC solubility depend on combustion conditions,^53^ which can be highly variable in household biomass stoves, making it difficult to distinguish between WSOC sources in these samples.

### Gender Differences in Personal Exposures

Across all three study sites, differences in P/O ratios by gender were generally small: men’s median P/O ratios for each species were 74–123% of women’s median P/O ratios among all but three of the chemical species included in the PCA analysis (Figure S7). Women had higher median P/O ratios than men for levoglucosan, picene, and WSOC (men’s median P/O ratios were 51, 57, and 67% of women’s, respectively). However, women’s and men’s median P/O ratios did not differ significantly for any of these species (Wilcoxon *p* > 0.05), indicating that variability in the P/O ratios within male and female subgroups was greater than between subgroups.

This overall similarity of chemical composition of men’s and women’s personal exposures in our subsample analysis supports our earlier finding from the full study population that men’s and women’s PM_2.5_ exposures were nearly identical after accounting for tobacco smoking.^32^ It is often assumed in HAP studies that women’s exposures exceed men’s due to more time spent cooking and inside the home.^7^ However, in our study of rural Chinese adults, although women were usually the primary cooks in their households, men were involved with heating stove use and other household energy tasks, and the majority of participants (>70%) at each study site had the same occupation.^32^ Additionally, in gender-specific mixed-effect models, use of an outdoor heating stove was associated with a smaller decrease in exposure for men than for women.^32^

Our results are similar to the few previous HAP studies comparing PM_2.5_ exposure and sources by gender, where women’s and men’s exposures did not differ significantly. In a multicountry study of rural communities using solid fuel stoves, exposures to PM_2.5_ were not different for men and women in China, India, Chile, Colombia, Tanzania, and Zimbabwe, despite women spending more time per day in the kitchen,^15^ although women in Bangladesh and Pakistan did have higher PM_2.5_ exposures than men.^15^ Men and women in households using biomass in peri-urban India had very similar PM_2.5_ exposures (means: 55.1 and 58.5 μg/m^3^, respectively), even though most men reported spending no time on cooking with biomass.^13^ In contrast, a small exposure study in rural Uganda and Ethiopia reported that women’s PM_2.5_ exposures were 5–6 times higher than men in the same villages.^29^ The spectrum of differences in gender-specific exposures highlights the importance of understanding the distribution of domestic responsibilities when assessing exposures, particularly if solid fuels are also used for heating in addition to cooking.

To our knowledge, no studies have assessed potential gender differences in the specific chemical components and sources of exposure in a setting of household solid fuel use. Chemical analysis of even a small subset of PM_2.5_ exposure samples, as was conducted in this study, can show whether a different set of sources contributes to women’s exposures compared to men’s exposures and thus explain trends observed in men’s and women’s overall PM_2.5_ exposure levels.

### Fuel Use and Exposure to Solid Fuel Tracers

To evaluate how PM_2.5_ exposures and fuel use were related, we compared P/O ratios of coal and biomass burning tracers in composites with versus without use of that fuel. Conceptually, P/O ratios of tracers of a given fuel should be much higher for users of that fuel than for those who do not use that fuel. Using P/O ratios rather than concentrations normalized to PM_2.5_ mass or air volume accounts for background ambient PM_2.5_ contributions, which also enables comparison across all study sites together.

**Figure 3:**
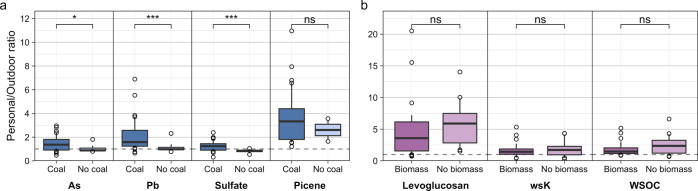
Personal/outdoor ratios of selected species emitted by (a) coal and (b) biomass burning. For each fuel, composites with use of that fuel are plotted as the darker box (left), while the lighter box (right) represents composites without use of that fuel. The dashed line is plotted at *y* = 1 for reference. Medians were compared using the unpaired Wilcoxon test (ns = not significant [*p* > 0.05]; * = *p* ≤ 0.05; and *** = *p* ≤ 0.001). Box-and-whisker statistics are calculated as described for Figure 2.

Coal users had higher P/O ratios of coal combustion tracers than participants not using coal (Figure 3a). For As, Pb, and sulfate, P/O ratios were greater than 1 for coal users and at or below 1 for nonusers, and differences were statistically significant (median coal and non-coal As: 1.4 and 0.9, *p* = 0.044; Pb: 1.6 and 1.0, *p* = 0.0008; and sulfate: 1.2 and 0.8, *p* = 0.0008). The same trend was observed for picene P/O ratios, although not statistically significant. Interestingly, all picene P/O ratios were greater than one, averaging 3.3 with coal use and 2.6 without coal use, likely due to coal use in nearby households which is not captured by ambient samples representing regional PM_2.5_.

Biomass burning tracers did not exhibit the expected trend: personal exposure composites with and without biomass users did not have significant differences in P/O ratios of levoglucosan, water-soluble K, and WSOC (Figure 3b). This may be partially attributable to these species’ selectivity as tracers. Water-soluble K and WSOC are emitted by biomass burning but are also components of dust and secondary aerosol, respectively.^53^ However, levoglucosan is a pyrolysis product of cellulose and thus considered specific to biomass burning,^82^ although it has also been detected at lower levels in emissions from coal combustion.^83,84^ Moreover, median P/O ratios of biomass tracers were greater than one regardless of biomass use (biomass and non-biomass levoglucosan: 3.6 and 5.9; wsK: 1.4 and 1.7; and WSOC: 1.5 and 2.4). These high levoglucosan P/O ratios, particularly for exposures among participants not using biomass, are evidence of exposure to biomass burning emissions that are neither from regional ambient PM_2.5_ nor participants’ own household fuel use. Most likely, as noted above for picene, it is proximity to other nearby homes using biomass stoves that elevates their exposures.

These discrepancies between self-reported fuel use and chemical components of actual exposures parallel discrepancies between fuel use and PM_2.5_ exposures or area concentrations, in this study and others. In our earlier study of all personal PM_2.5_ exposures, exclusive clean fuel users had less than 15% lower PM_2.5_ exposure compared with solid fuel users, even after adjusting for outdoor PM_2.5_ and other sociodemographic variables.^32^ A different source apportionment study of personal PM_2.5_ exposures in Yunnan province (China) found that, although cooking and wood combustion typically accounted for over 75% of women’s PM_2.5_ exposures, self-reported cooking frequency was not strongly correlated with contributions from either source.^30^ Duration of wood-chimney stove use was also not a predictor of kitchen PM_2.5_ in a study in rural households in Sichuan, China.^31^

The importance of community-level change has been noted for some of the many complex factors in household energy transitions (such as adoption and continued use) and increasingly also for achieving meaningful reductions in air pollution exposures in order to improve health outcomes.^85,86^ We found that chemical tracers of solid fuel combustion were present in exposures at levels not entirely explained by household fuel use or background levels in outdoor PM_2.5_, indicating that fuel use in neighboring homes may impact personal exposures. Notably, exposures of participants using biomass were not higher in levoglucosan than those of participants not using biomass, even after accounting for background ambient levels. Chemical composition of PM_2.5_ exposures did not differ between men and women, despite differences in domestic activities. Our findings contribute to growing evidence that reducing exposures to HAP will require both household- and community-level changes in stove and fuel use and that outdoor air pollution is an important consideration when evaluating or predicting the efficacy of household-level air pollution controls.

## Supporting Information Available

Additional details about study sites, sample selection, and fuel use data; compositing scheme, fuel use trends, filter and blank PM_2.5_ masses, picene/hopane comparison, and personal/outdoor ratios of chemical components; and summary statistics for chemical species and source contributions (PDF)
