# Supplementary material for: Chemical Investigation of Household Solid Fuel Use and Outdoor Air Pollution Contributions to Personal PM2.5 Exposures
Source: Environ Sci Technol. 2021 Nov 24;55(23):15969–79. doi: 10.1021/acs.est.1c01368 (PMC8655976; doi:10.1021/acs.est.1c01368)
Supplement: Supplementary file 1 — es1c01368_si_001.pdf [file es1c01368_si_001.pdf]

Supporting Information for

A chemical investigation of household solid fuel use and outdoor air pollution contributions to personal $PM_{2.5}$ exposures

Alexandra Lai, Martha Lee, Ellison Carter, Queenie Chan, Paul Elliott, Majid Ezzati, Frank Kelly, Li Yan, Yangfeng Wu, Xudong Yang, Liancheng Zhao, Jill Baumgartner, James J. Schauer

Contents:

- Figure S1. Schematic of outdoor and personal exposure sample composites for chemical analysis
- Section S1. Study sites
- Section S2. Sample selection and fuel use summary
- Figure S2. Household fuel use patterns in composite samples selected for chemical analysis
- Figure S3. Summary of blank filter masses (µg) by site and season
- Figure S4. $PM_{2.5}$ concentrations of individual samples selected to composite for chemical analysis
- Figure S5. Mass comparison of samples selected for chemical analysis with all study samples
- Table S1**.** Summary statistics for chemical component concentrations (ng/m^3 or µg/m^3)
- Table S2. Summary statistics for mass-normalized source contributions (g/g PM)
- Figure S6. Scatterplot of picene and hopane concentrations in personal exposure $PM_{2.5}$ samples at each site
- Figure S7. Personal/outdoor ratios of $PM_{2.5}$ chemical components, grouped by gender

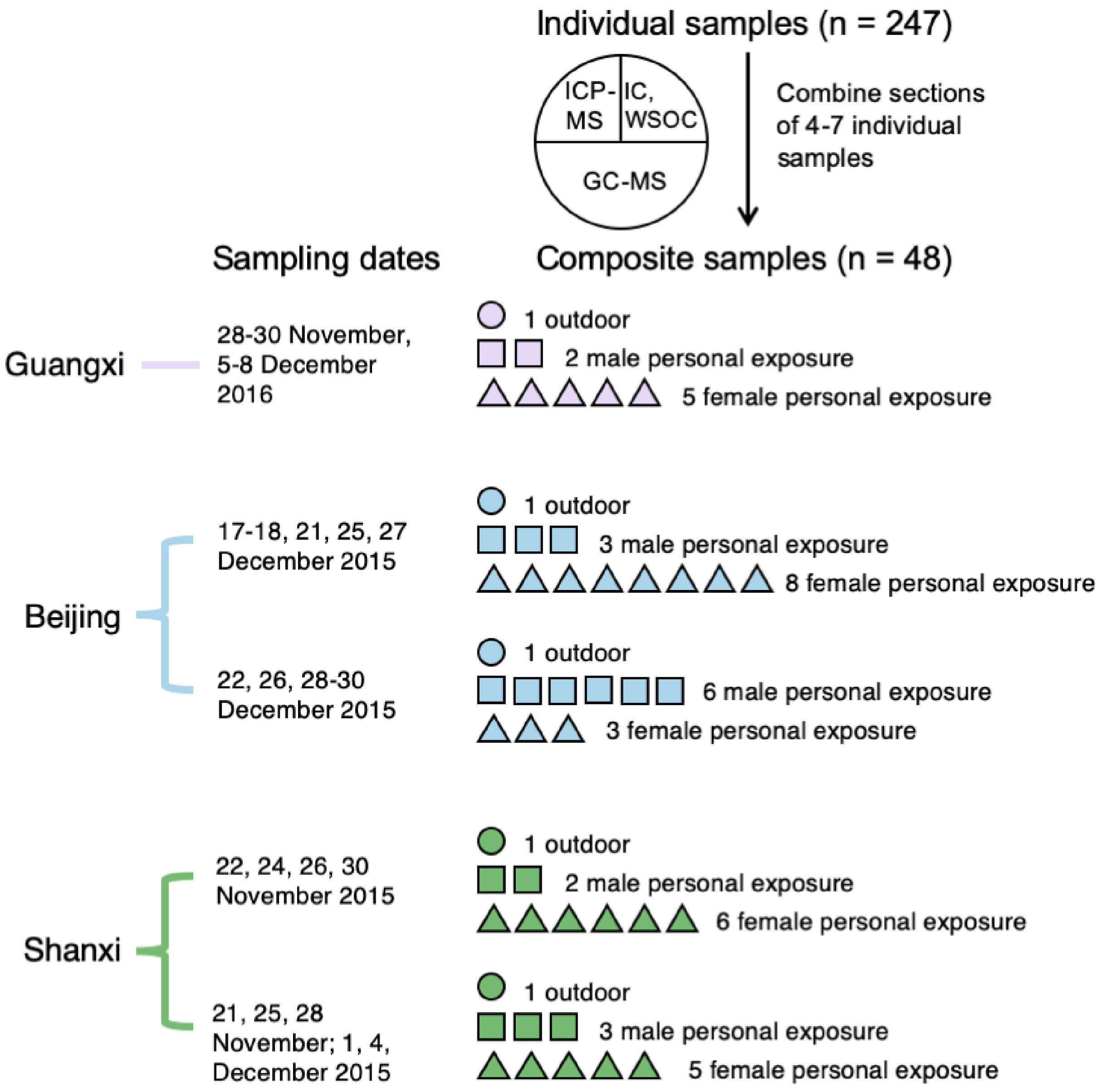

Figure S1. Schematic of outdoor and personal exposure sample composites for chemical analysis. Each composite consists of 4-7 individual samples, corresponding to the dates for that set of composites. IC = ion chromatography; WSOC = water-soluble organic carbon, ICPMS = inductively coupled plasma mass spectrometry; GC-MS = gas chromatography-mass spectrometry

Section S1. Study sites

At field sites in Shanxi and Beijing, most households used biomass, crop residues, and coal for cooking and coal for indoor space heating in winter. Guangxi households used wood and crop residues as cooking fuel and do not require winter heating. While households from all three sites use liquefied petroleum gas (LPG) for cooking, some households in Shanxi and Beijing continue to also use biomass and/or coal for cooking, and more than half continue to use primarily solid fuels for heating. At all three sites, participants' villages are becoming less rural and more peri-urban, including increased presence of local industry, density of road networks, and associated vehicle traffic. Potential local industry sources include construction, food processing plants, and manufacturing (e.g. pharmaceuticals, machine parts, automobiles, plastics), many of which are coal-powered.

Section S2. Sample selection and fuel use summary

To assess fuel use, we developed visual libraries of stoves and fuels for each study site. For each stove pictured, participants indicated whether they currently used this stove along with intensity of use, location of the stove, and fuel. Use of stoves/fuels was assessed for both cooking and, where applicable (Shanxi and Beijing), heating. The use of clean fuels included gas and electricity, while solid fuel included coal, wood, crop residues and other forms of biomass. Field staff visited a random subsample of homes in each village to assess accuracy in self-report. If government records of infrastructure change were available (e.g. installation of gas lines), survey data were also cross-referenced with these records.[1] Additional detailed information on questionnaire development to assess current fuel and stove use is available elsewhere.[1]

All selected samples were collected in winter: November/December 2015 (Beijing and Shanxi) and November/December 2016 (Guangxi). Each composite had quarter sections of between 4 and 7 samples. Only samples with sampling times within +/- 20% of the target sampling time (24 hours) were included in composites. Each set of composites (outdoor and male and female personal exposure) corresponded to the same set of 4-7 dates, so that outdoor and personal PM exposures are directly comparable within each set (Figure S1). We excluded personal $PM_{2.5}$ exposure samples from active smokers to avoid tobacco smoke contributions to personal exposures. The 221 personal exposure sample were from 171 individuals, so there were multiple samples from some participants, but participants were not repeated within composites, i.e. no composite contained more than one sample from a given individual. Fuel use data was not available for ~2% of the individual samples selected for chemical analysis; excluding those, 41 of the 43 personal exposure composites had complete fuel use data.

Selected samples at the three sampling sites had distinct energy profiles in terms of participants' use of coal, biomass, and clean fuels (Figure S2). Fuel use in households selected for chemical analysis followed similar trends to those of the study as a whole,[1] though with more mixed fuel use as a result of combining individual samples into composites. Personal exposure composites were differentiated as much as possible in terms of fuel use, but within the constraints of choosing concurrent personal and ambient samples, some loss of resolution of fuel use data was unavoidable. Clean fuels (LPG and/or electricity) were used by households in all composites, but only Guangxi had composites with exclusive clean fuel use (n = 2). The remaining Guangxi composites included both biomass and clean fuel use, and there was no coal use in Guangxi. Coal use was highest in Shanxi, where all households in all composites used both coal and clean fuels; about half of these (7 of 16) also used biomass. All three fuels were used in the majority of Beijing composites (15 of 20). Of the remaining five Beijing composites, two used biomass and clean fuels, one used coal and clean fuels, and fuel use data was not available for two.

The fuel use patterns observed for these samples and the study overall are somewhat consistent with trends at the province level. A recent survey of fuel use in China reported that coal was the primary household energy source for more than 80% of households in Shanxi province; in Beijing, just over half of households used gas and approximately one-third used biomass; and biomass was the main fuel for about two-thirds of households in Guangxi.[2] Note that the fuel use data collected for our samples was whether each fuel type was or was not used, regardless of frequency or preference, while the survey data presented by Duan et al.[2] presumably represents households' primary fuel but does not specify if fuel use was exclusive or mixed.

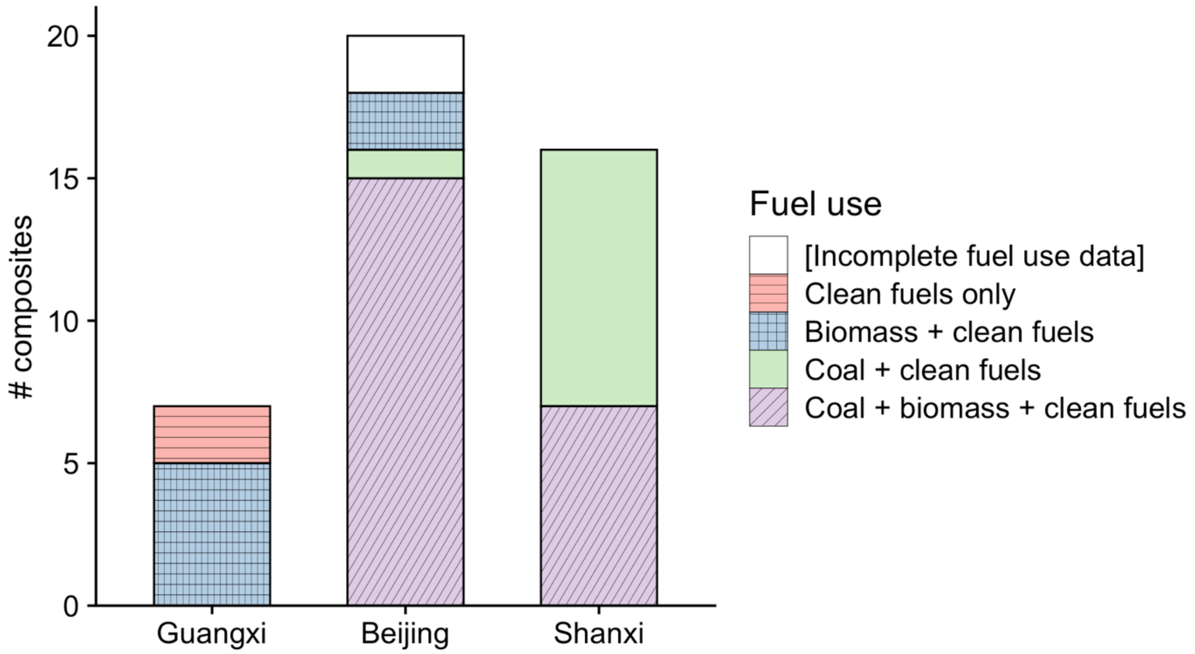

Figure S2. Household fuel use patterns in composite samples selected for chemical analysis. Heating fuel and cooking fuel data are combined. Fuel use reported here represents the composite as a whole, e.g. a composite with coal and clean fuel use may include individual samples from households using coal or clean fuels exclusively.

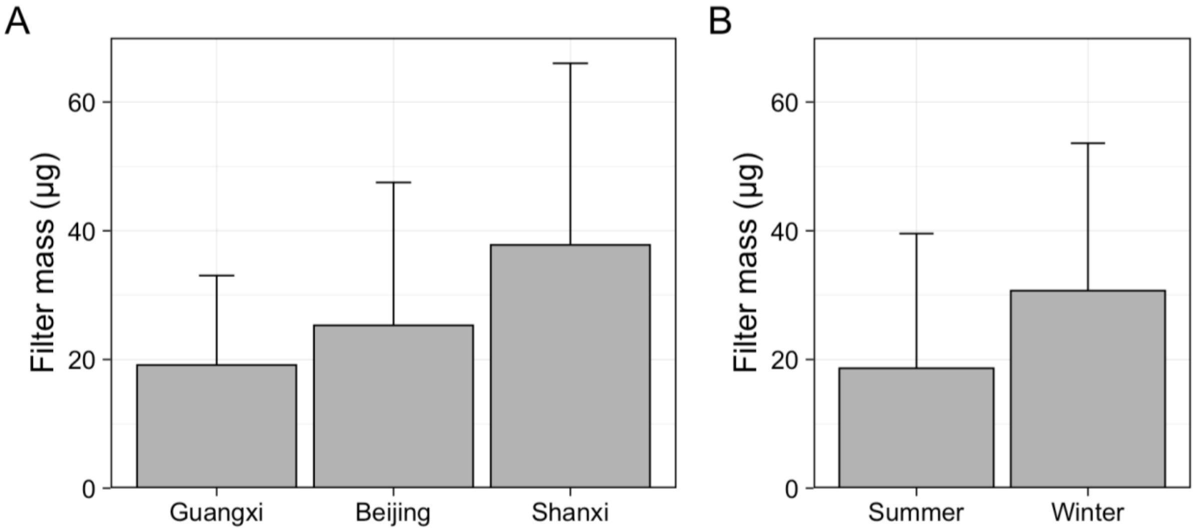

Figure S3. Summary of blank filter masses (μg) by site and season. Bar height and error bars indicate arithmetic mean ± standard deviation.

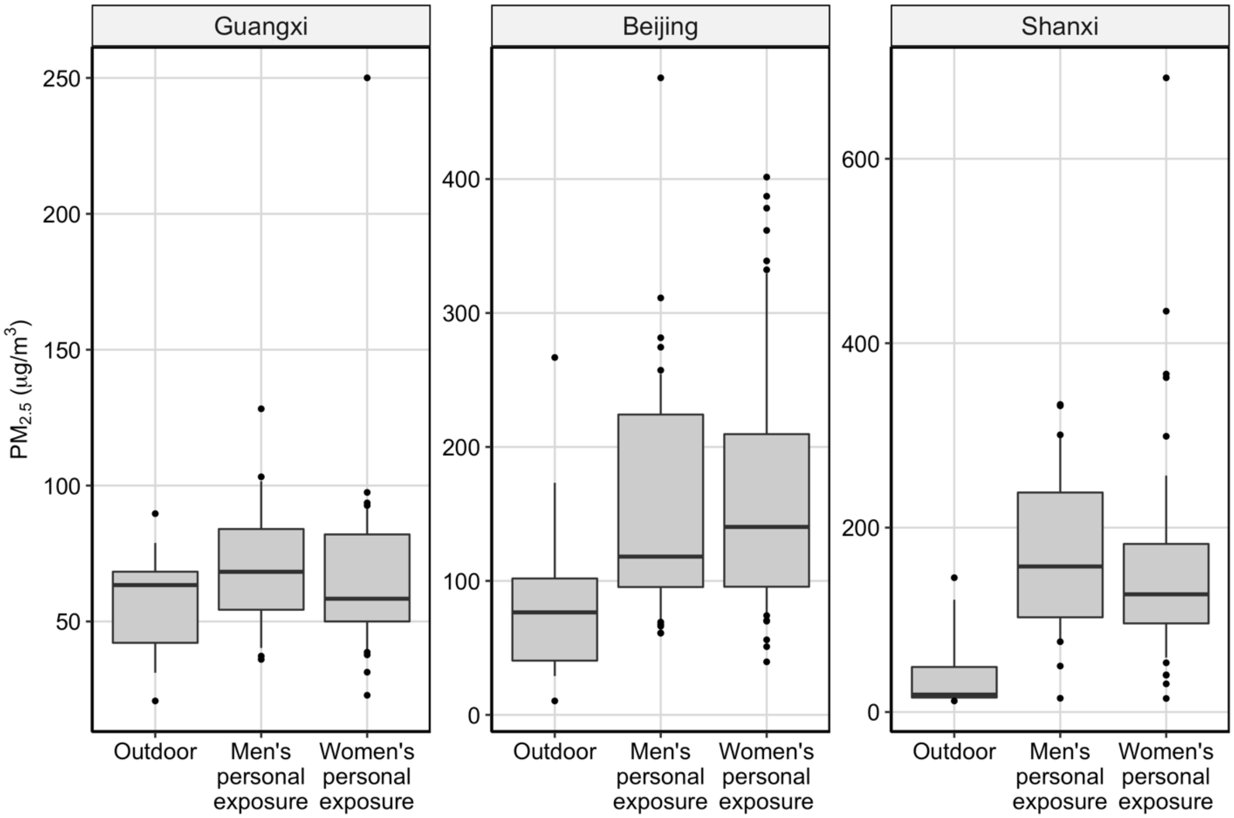

Figure S4. $PM_{2.5}$ concentrations of individual samples selected to composite for chemical analysis. Sample sizes are available in Table 1. Box midlines indicate median ratios, the box represents the interquartile range (IQR; top = 75th percentile; bottom = 25th percentile), and whiskers extending above and below the box mark the 90th and 10th percentiles, respectively. Individual points represent values that were above the 90th percentile or below the 10th percentile.

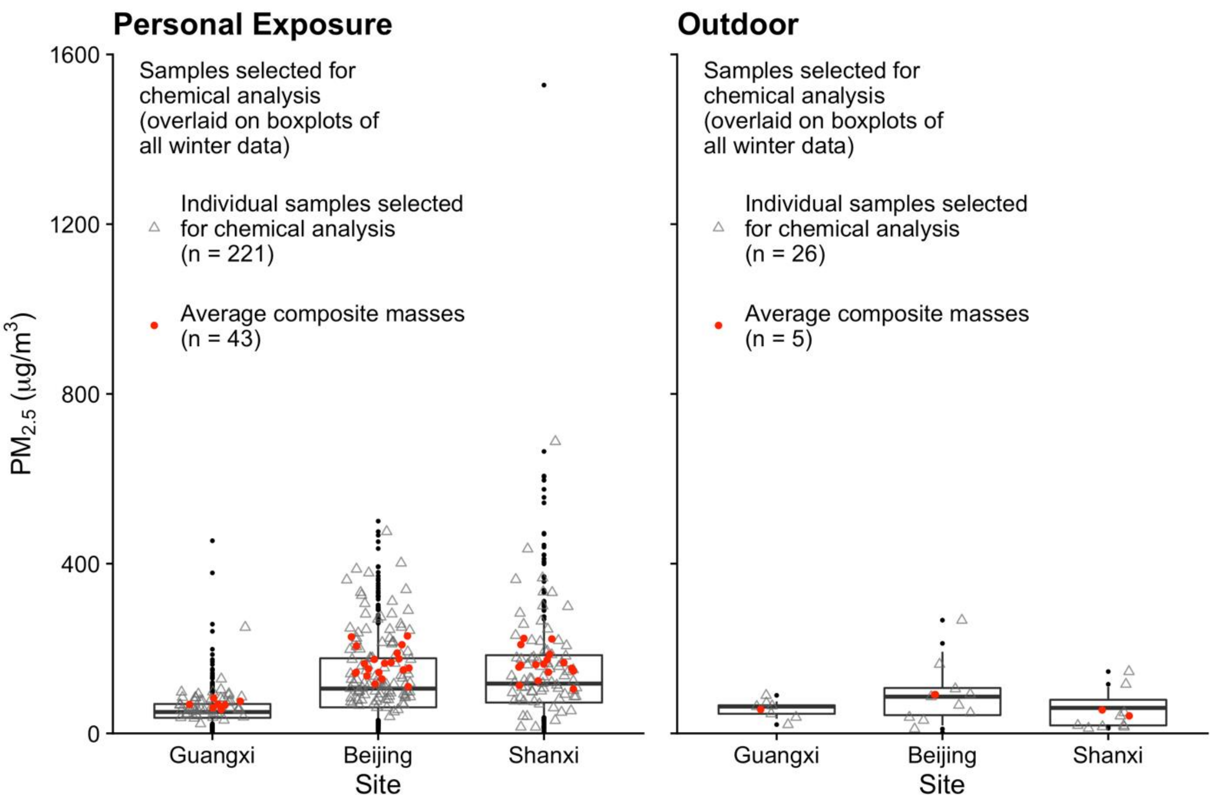

Figure S5. Mass comparison of samples selected for chemical analysis with all study samples, which are presented in Lee et al. (2021).[3] Box statistics are calculated as described for Figure S4.

Table S1. Summary statistics for chemical component concentrations (ng/m^3 or µg/m^3[*])

	Outdoor					Personal					
	Guangxi§	Beijing		Shanxi		Guangxi		Beijing		Shanxi	
		Mean	SD	Mean	SD	Mean	SD	Mean	SD	Mean	SD
*WSOC	5.8	6.5	0.3	3.4	0.4	6.2	1.3	10.6	4.1	10.3	4.7
*Na^+	0.3	0.3	0.2	0.2	0	0.1	0.1	0.4	0.2	0.2	0.2
*NH_4^+	2.2	7.9	0.6	7.1	3.1	1.5	0.4	13	9.4	10.1	5.7
*K^+	1.3	0.6	0	0.3	0.1	0.6	0.1	0.9	0.3	0.7	0.3
*Cl^-	0.3	0.9	0.3	0.3	0.2	0.2	0.1	1.8	0.9	2.4	2.5
*NO_3^-	0.3	1.9	0.4	0.4	0.2	0.2	0.2	4.8	6.9	1.2	1.9
*SO_4^{2-}	6.3	9.5	0.3	12.7	5.1	4.8	0.8	11.5	3.6	16.6	8.4
Li	0.5	1.1	0.5	2	0.5	0.5	0.2	1.7	0.5	2.5	0.8
B	56.2	32.7	4.3	9.5	1.7	59.8	4.6	82.2	23.4	45.2	17.3
Na	395	416.9	185.4	72.8	9.6	334.4	157.5	516.1	207.9	118.5	85.8
Mg	102	99.9	24.2	26.3	17.2	130.2	86.6	225.3	94	77	47.4
P	17.1	19.1	2.7	55.2	52.6	36.3	30.7	79.4	93.3	51.2	20.6
K	1181.1	755.2	44.4	221.9	4.5	856.8	162	1266.7	381.4	677.4	275.9
Ca	670.3	246.7	8.1	78.1	13.5	2228.4	3184.1	642.9	378.9	325.9	162.7
Ti	29.4	13	3.3	7.4	0.3	36.2	10	26.2	12.2	28.6	12.6
V	1.1	1	0	0.5	0.1	1.4	0.3	1.7	0.6	1	0.3
Mn	26.3	25.3	3.1	7.7	3.4	28	6.7	42.3	12.5	17.5	8.7
Fe	254	330.1	60.3	111.5	58.2	362.8	73.9	518.9	251	165.7	41.8
Zn	44.1	96.1	0.2	64.1	38.6	55.2	22.3	159.4	66	127.7	73.9
As	6	5.9	0.8	6.7	2.5	5.6	0.8	7.9	2.8	10.3	5.2
Rb	4	2.2	0.3	1	0.1	2.2	0.4	3.6	1.6	1.7	0.5
Sr	2.3	2.3	0.2	1.5	0.2	3	1.8	4.7	2.6	5.1	2.6
Y	0.1	0.1	0	28.3	40	0.2	0.1	0.2	0.1	0.2	0.1
Ag	0.1	0.2	0	0.1	0.1	0.4	0.6	0.3	0.2	0.1	0.1
Cd	1.4	1.1	0	0.8	0.6	1.5	0.3	2.3	1.1	2.4	0.9
Sb	7.3	3	0.6	1.1	0.3	5.7	0.7	19.8	70	3.1	2.4
Cs	0.3	0.3	0.1	0.1	0	0.3	0	0.5	0.2	0.2	0.1
Ba	7.5	6.2	0.5	2.2	0.1	7.7	1.7	8.5	2.5	5	1.7
La	0.17	0.179	0.036	0.12	0.026	0.27	0.10	0.31	0.13	0.39	0.18
Ce	0.30	0.28	0.043	0.23	0.057	0.49	0.18	0.52	0.25	0.73	0.33
Pr	0.033	0.023	0.002	0.023	0.005	0.052	0.022	0.05	0.027	0.075	0.034
Nd	0.12	0.09	0.001	0.079	0.017	0.19	0.079	0.18	0.1	0.26	0.11
Sm	0.019	0.014	0.001	0.014	0.003	0.034	0.015	0.032	0.019	0.049	0.023
Eu	0.006	0.004	0	0.003	0	0.008	0.003	0.009	0.004	0.01	0.004
Dy	0.021	0.014	0	0.013	0.003	0.031	0.012	0.029	0.016	0.043	0.021
Ho	0.004	0.002	0	0.003	0.001	0.006	0.002	0.006	0.003	0.009	0.004
Yb	0.014	0.006	0.001	0.008	0.002	0.019	0.007	0.015	0.009	0.024	0.012
Lu	0.002	0.001	0	0.001	0	0.003	0.001	0.002	0.001	0.004	0.002
Hg	0.1	0.3	0.1	0.6	0.1	0.5	0.3	1.2	1.1	2.2	1.1
Tl	0.4	0.9	0.1	0.3	0	0.4	0	1.7	0.9	0.6	0.2
Pb	37.2	56.4	1.4	42.3	11	36.3	5.1	95.6	44	112.9	75.2
Th	0.1	0	0	0	0	0.1	0	0.1	0	0.2	0.1
U	0	0	0	0.1	0.1	0	0	0.1	0	0.2	0.1
Levoglucosan	373.5	770.3	383.0	217.5	58.9	901.9	390.7	1982.5	1201.0	1714.0	741.1
Benzo[*b*]fluoranthene	1.2	29.8	2.9	22.9	16.3	2.6	0.7	57.9	15	70.6	55.6
Benzo[*k*]fluoranthene	0.3	15.2	1.6	5.1	3.1	1.3	0.3	24.8	10.3	13.8	5.8
Benzo[*j*]fluoranthene	0	1.6	0.7	0.4	0.2	0	0	3.1	1.5	1	0.7
Benzo[*e*]pyrene	0.8	17.1	4.9	18.8	14	1.8	0.4	30.2	8.5	72.3	75.4
Benzo[*a*]pyrene	0.7	11.6	5.1	2.8	1.3	1.2	0.3	20.1	7.6	12.8	8.7
Perylene	0	1.8	0.9	0.5	0.2	0	0	3.3	1.4	1.3	0.7
Indeno[1,2,3-*cd*]pyrene	0	18.4	2.3	6.7	3.6	2.6	0.7	33.3	8.7	21.3	10.6
Benzo[*ghi*]perylene	1.5	15.2	2.5	14	9.6	2.6	0.5	26.8	7	52.9	38.7
Dibenz[*ah*]anthracene	0	2.5	0	3.7	2.7	0.2	0.5	5.1	1.2	14.4	8.3
Picene	0	2.3	0.5	4.2	3.2	0	0	5.9	2.2	15	9.3
Coronene	0	3	0.2	3.3	2.3	1	0.1	5.6	1.5	12.6	8.4
Dibenzo[*ae*]pyrene	0	2.2	0.1	6.5	5.5	0	0	4.7	0.9	27.7	22.5

***** Species marked with an asterisk are in units of µg/m^3; all other data is in ng/m^3

§ Only one composite of Guangxi outdoor samples was analyzed; shown here are values for that composite

Table S2. Summary statistics for mass-normalized source contributions (g/g PM)

Site	Sample type	Summary statistic	Biomass burning	Coal combustion	Dust	Secondary ammonium	Secondary nitrate	Secondary sulfate	Vehicles
Guangxi	Outdoor[1]	--	0.08	0	0.09	0.04	0	0.11	0.04
	Men	Median	0.2	0	0.12	0.02	0	0.06	0.02
		Mean	0.2	0	0.12	0.02	0	0.06	0.02
		Std. Dev.[2]	0.02	0	0.02	0	0	0	0.01
		Min	0.19	0	0.1	0.02	0	0.06	0.01
		Max	0.22	0	0.13	0.02	0	0.06	0.03
	Women	Median	0.12	0	0.18	0.02	0	0.08	0.02
		Mean	0.13	0	0.18	0.02	0	0.07	0.02
		Std. Dev.	0.06	0	0.08	0.01	0	0.01	0.01
		Min	0.08	0	0.07	0.02	0	0.06	0.01
		Max	0.22	0	0.29	0.03	0.01	0.09	0.04
Beijing	Outdoor	Median	0.1	0.1	0.02	0.09	0.02	0.09	0.12
		Mean	0.1	0.1	0.02	0.09	0.02	0.09	0.12
		Std. Dev.	0.05	0.02	0.01	0.01	0	0.01	0.01
		Min	0.06	0.08	0.02	0.08	0.02	0.08	0.11
		Max	0.13	0.11	0.03	0.09	0.02	0.09	0.12
	Men	Median	0.1	0.12	0.09	0.05	0.01	0.05	0.09
		Mean	0.11	0.14	0.1	0.09	0.03	0.05	0.11
		Std. Dev.	0.05	0.05	0.04	0.09	0.07	0.02	0.03
		Min	0.07	0.08	0.06	0.04	0.01	0.02	0.07
		Max	0.22	0.24	0.17	0.34	0.22	0.08	0.16
	Women	Median	0.15	0.13	0.08	0.06	0.01	0.04	0.1
		Mean	0.16	0.14	0.09	0.08	0.03	0.05	0.13
		Std. Dev.	0.09	0.06	0.03	0.04	0.02	0.02	0.06
		Min	0.06	0.07	0.05	0.04	0.01	0.02	0.09
		Max	0.39	0.27	0.15	0.15	0.07	0.08	0.27
Shanxi	Outdoor	Median	0.05	0.29	0.01	0.14	0.01	0.22	0
		Mean	0.05	0.29	0.01	0.14	0.01	0.22	0
		Std. Dev.	0	0.18	0	0.04	0	0.03	0
		Min	0.05	0.16	0.01	0.12	0.01	0.2	0
		Max	0.05	0.41	0.01	0.17	0.01	0.25	0
	Men	Median	0.12	0.28	0.04	0.03	0	0.05	0
		Mean	0.12	0.28	0.05	0.05	0.01	0.05	0
		Std. Dev.	0.07	0.09	0.04	0.04	0.01	0.05	0
		Min	0.05	0.17	0.01	0	0	0	0
		Max	0.22	0.39	0.11	0.1	0.03	0.13	0
	Women	Median	0.12	0.3	0.04	0.07	0	0.06	0
		Mean	0.13	0.35	0.03	0.07	0.01	0.07	0
		Std. Dev.	0.06	0.21	0.02	0.03	0.02	0.03	0
		Min	0.04	0.07	0	0.02	0	0.03	0
		Max	0.25	0.86	0.09	0.11	0.04	0.12	0

[1] Only one outdoor composite was analyzed for Guangxi

[2] Standard deviation

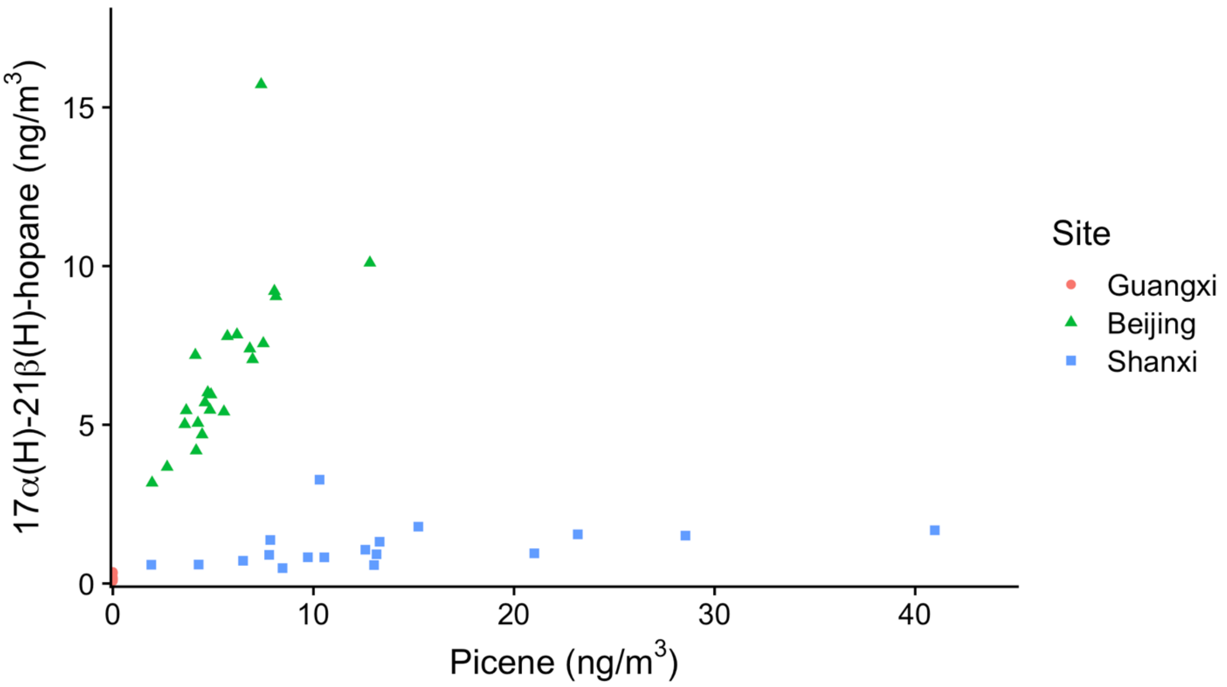

Figure S6. Scatterplot of picene and hopane concentrations in personal exposure $PM_{2.5}$ samples at each site.

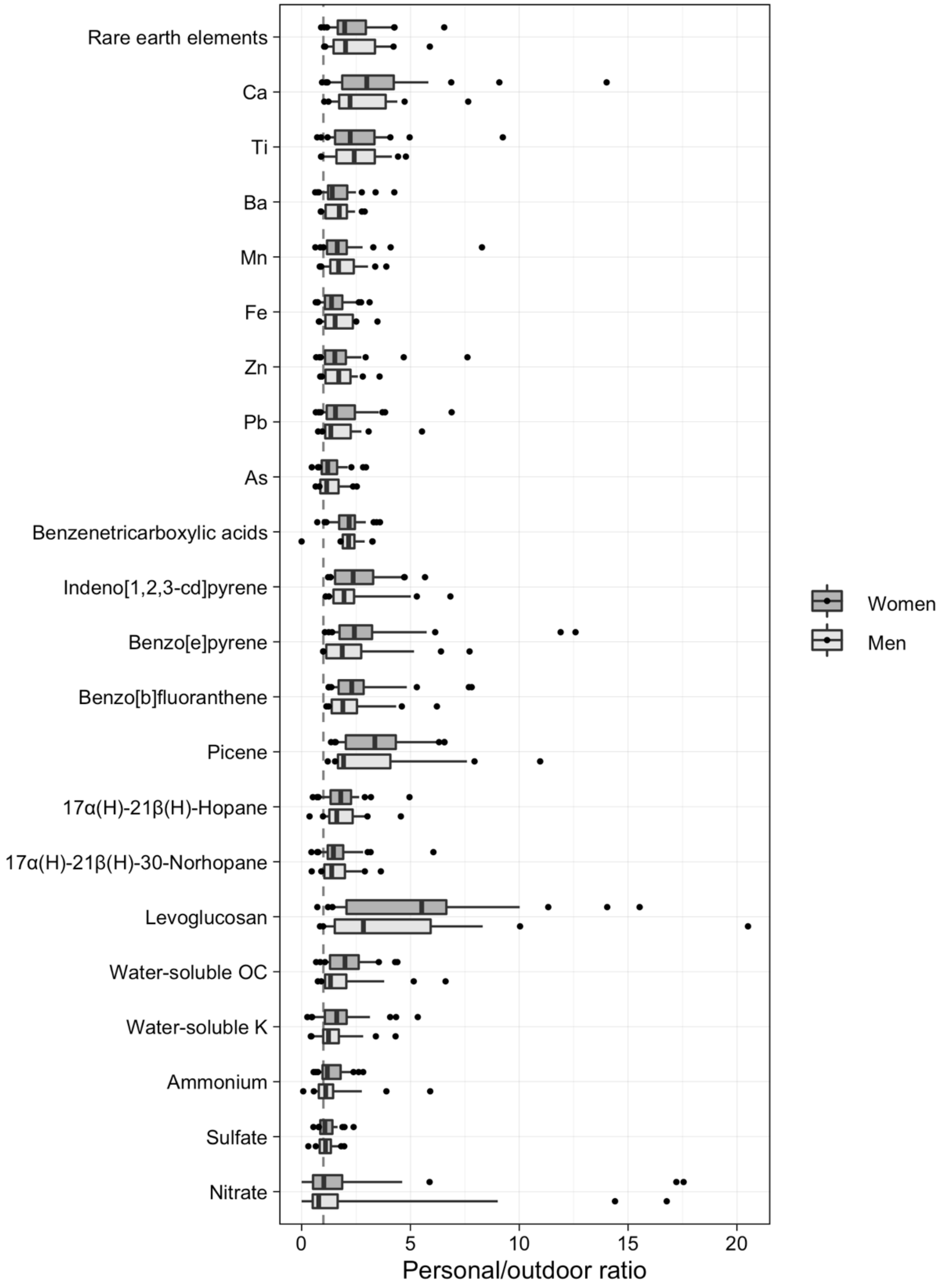

Figure S7. Personal/outdoor ratios of $PM_{2.5}$ chemical components, grouped by gender. Box statistics are calculated as described for Figure S4. Dashed line is plotted at x = 1 for reference.

References

(1) Carter, E.; Yan, L.; Fu, Y.; Robinson, B.; Kelly, F.; Elliott, P.; Wu, Y.; Zhao, L.; Ezzati, M.; Yang, X.; Chan, Q.; Baumgartner, J. Household Transitions to Clean Energy in a Multiprovincial Cohort Study in China. *Nat Sustain* **2020**, *3* (1), 42–50. https://doi.org/10.1038/s41893-019-0432-x.

(2) Duan, X.; Jiang, Y.; Wang, B.; Zhao, X.; Shen, G.; Cao, S.; Huang, N.; Qian, Y.; Chen, Y.; Wang, L. Household Fuel Use for Cooking and Heating in China: Results from the First Chinese Environmental Exposure-Related Human Activity Patterns Survey (CEERHAPS). *Applied Energy* **2014**, *136*, 692–703. https://doi.org/10.1016/j.apenergy.2014.09.066.

(3) Lee, M.; Carter, E.; Yan, L.; Chan, Q.; Elliott, P.; Ezzati, M.; Kelly, F.; Schauer, J. J.; Wu, Y.; Yang, X.; Zhao, L.; Baumgartner, J. Determinants of Personal Exposure to PM2.5 and Black Carbon in Chinese Adults: A Repeated-Measures Study in Villages Using Solid Fuel Energy. *Environment International* **2021**, *146*, 106297. https://doi.org/10.1016/j.envint.2020.106297.
